# Condensation Effect and Transport on Alumina Porous Membranes

**DOI:** 10.1021/acs.langmuir.4c04606

**Published:** 2025-06-20

**Authors:** Fernanda R. Leivas, Menghua Zhao, Aymeric Allemand, Cécile Cottin-Bizonne, Stella M. M. Ramos, Marcia C. Barbosa, Anne-Laure Biance

**Affiliations:** † Université de Lyon, Université Claude Bernard Lyon 1, CNRS, Institut Lumière Matière, F-69622 Villeurbanne, France; ‡ Universidade Federal do Rio Grande do Sul, Instituto de Física, CP 15051, 91501-970 Porto Alegre, Rio Grande do Sul, Brazil

## Abstract

Understanding the adsorption of water and characterizing the water film formed within nanostructures are essential for advancements in fields such as nanofluidics, water purification, and biosensing devices. In our research, we focused on studying the condensation and transport of water through an alumina membrane with nanopores of varying wettabilities. We introduce a method to alter the membrane’s wettability and enhance dissociative adsorption by varying the duration of exposure during plasma cleaning. To create different experimental environments, we modify humidity levels by controlling the vapor pressure. To investigate water transport within the membrane, we apply a voltage and analyze the resulting current response. Our analysis indicates that transport properties improve with thicker water films. We used the Polanyi theory of adsorption to capture the physics of the problem. Analyzing the conductance inside the nanopores, we find that the first monolayers may stagnate due to interactions with the pore walls. This research significantly enhances our understanding of vapor condensation within nanomaterials, particularly considering the influence of different wettabilities. These findings have broad implications for applications such as water vapor capture and related technologies.

## Graphical abstract

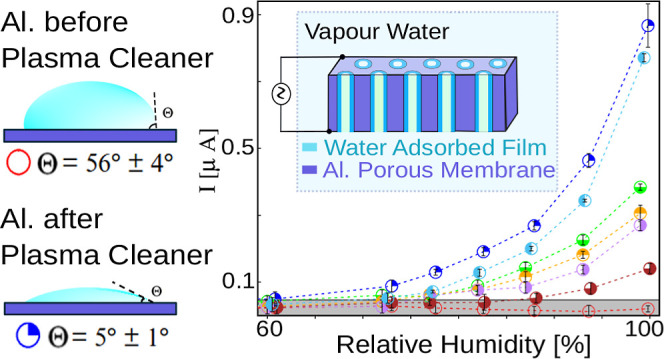

## Introduction

The characterization of water film adsorption and transport within nanopores is fundamental to understanding many physical issues such as water purification,^1–4^ energy storage,^5,6^ and geological^7^ and biological problems such as drug delivery or chemical sensing.^8–12^ The process of adsorption varies depending on factors such as temperature, humidity, and material properties like wettability, roughness,^13–15^ and the hydrophobicity.^16–18^ Condensation and transport of water at the nanoscale can then be useful for developing atmospheric water harvesting with a low energetic cost.^19,20^

The vast area of nanofluidics has been attracting the attention of researchers in recent years.^12,21–23^ Nanoconfined water is organized in layers,^24^ with the contact layer being more structured^25^ and exhibiting slower diffusional dynamics compared to those further from the surface.^26^ In this multilayer structure, the slip length is a fundamental parameter that controls the flow of confined water under huge pressures.^27^ Although the heterogeneous dynamics of confined waters under pressure is well established, there is still a need for precise quantification of the layer thickness and its mobility at different levels of wettability.^28–32^

A convenient way to study the water mobility is the electrokinetic method that we adopted in the current study. In this case, an external electrical field is applied, and the subsequent current is measured, from which we deduce the ion mobility and the water flow. Because of the strong interactions between the liquid and surface at the interface,^24,25,33^ the nature of the confining material is expected to affect this process. Allemand et al.^34^ investigated nanoscale transport properties on a silica (SiO_2_) surface, controlling water layer thickness by adjusting relative humidity (RH). They proposed a new method to explore ionic transport under extreme molecular confinement by adjusting liquid film thickness from 0.3 to 2 nm. Their findings during conductance measurements revealed anomalous ionic transport, indicating the presence of a stagnant hydrodynamic layer in very thin liquid films, which is consistent with the low mobility of the contact layer of water under pressure. In addition to SiO_2_, alumina (Al_2_O_3_) is a versatile oxide widely used as ceramic membranes due to its notable properties, including high mechanical and chemical stability, good permeation performance, and electrical properties.^35–41^

In the particular case of the Al_2_O_3_, the charge behavior and molecular orientation in the adsorbed liquid can vary depending on the pH.^42,43^ While Al_2_O_3_ substrates exhibit a layer-by-layer adsorption process (physisorption), single crystals feature hydroxyl groups (chemisorption).^44^ It has been reported that the α-Al_2_O_3_ surface, the most stable form of aluminum oxide, shows either no dissociative adsorption or a very slow process in contact with water under ambient conditions.^45,46^ In contrast, other studies report the formation of hydroxyl groups and protonation at the interface.^47–49^ According to Tong and co-workers,^50^ α-Al_2_O_3_ surfaces are unreactive with respect to water adsorption, but the additional surface treatment, such as oxygen plasma cleaning, can alter surface adsorption properties.^47^

In order to circumvent the inconvenience of the presence of contaminants, the system can be submitted to the process of plasma cleaning, which involves the interaction of ions with a surface to remove undesirable molecules. In the meantime, the ionized oxygen activates some sites,^51–53^ and renders the surface hydrophilic through oxidation.^54,55^ Such a cleaning protocol on α-Al_2_O_3_ surfaces results in very low contact angles.^56^ Plasma cleaning is believed to facilitate dissociative adsorption at the α-Al_2_O_3_/water interface. The plasma cleaning, therefore, can be used to control the wettability of the system.

In this article, we provide a quantitative analysis of the water mobility for different wettabilities. The water adsorption and transport properties confined in Al_2_O_3_ nanoporous membranes are studied under varying conditions of wettability and RH. The contact angle was controlled by adjusting the exposure time of the plasma cleaning treatment. The experiment was carried out inside a vacuum chamber, and RH is controlled by adjusting vapor pressure. Ionic transport in water was studied by monitoring the current response when applying a sinusoidal voltage difference of amplitude 0.8 V.

Here, we introduced a novel application of plasma cleaning to control wettability and induce dissociative adsorption on a relatively nonreactive surface such as Al_2_O_3_ porous membranes. By applying a potential difference across the membrane, we discovered that the current response of the film formed within the pores is more pronounced at higher levels of hydrophilicity and RH. This suggests an enhanced transport as the film thickness increases.

Additionally, we observed that Polanyi’s theory^57^ effectively explains the physics involved, revealing an increase in adsorption energy with greater hydrophilicity. The use of the electric field instead of a pressure allows us to investigate the conductivity and conductance as a function of the thickness of the water layer.

The remainder of the article proceeds as follows: the next part presents the Methods employed in this study, followed by the Results and Discussion. Conclusions finalize the manuscript.

## Materials and Methods

### Membrane Properties

Anodisc aluminum oxide membranes of 55 μm thickness purchased from Sigma-Aldrich were used as the substrate. The substrates were pierced by nanometric conical pores, randomly distributed, with diameters of 200 and 100 nm (on the opposite side) and a mean interpore distance of 320 nm. These geometrical characteristics lead to a porous fraction, defined as the ratio between pore area and total membrane area, of 0.3 on the 200 nm side.

To perform conductivity measurements throughout the membrane, a thin Pt film of 20 nm thickness was deposited with an electron gun evaporator (Alliance Concept-model EVA300) on the surface with the larger 200 nm pores without causing any obstructions inside the pores (Figure 1b). The number of pores, *N*_p_, surrounded by Pt (on the metalized zone) is determined by the nanopore density multiplied by the corresponding area ≈1.8 cm^2^, *N*_p_ = 1.7 × 10^9^ ± 0.1.

**Figure 1:**
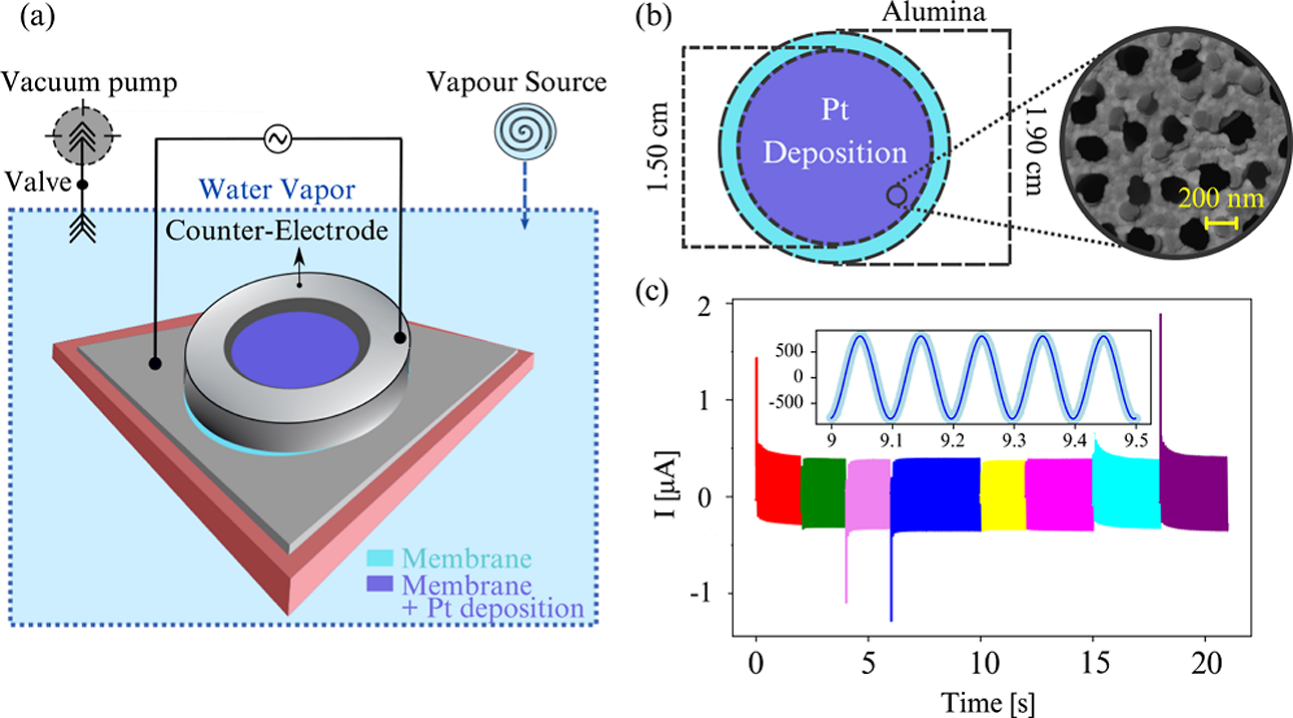
(a) Scheme illustrating the setup used for conductance measurement under controlled vapor pressure. (b) Scheme and SEM picture of the Anodisc alumina membrane. Black spots correspond to nanoporous zone. The membrane diameter is 1.9 cm, and a metallic deposition, gray zone on SEM image, was applied with a diameter of 1.5 cm. The nanopores are randomly distributed with an average distance of 320 nm. (c) Electrical current measured as a function of time (RH = 90%, Θ = 5° ± 1°). The input signal was transmitted and interrupted eight times, represented on the graph by different colors. Inset: time zoom of the current response *I* (μA) as a function of time. The black line corresponds to a sinusoidal fit, *I*(*t*) = *A* sin­(20π*t* + θ) + *c*. The resulting current is determined as the average of the resulting amplitudes (*A*) from these eight fits.

Roughness measurements were conducted by using an atomic force microscope (AFM, Asylum Research, MFP3D) operating in tapping mode. A zone of 4 μm^2^ was scanned on both sides of the membrane; the RMS roughness, defined as the average height deviations across a surface, was found to be ≈50 nm on the 200 nm side and ≈450 nm on the 100 nm side. To probe the roughness inside the pore, one sample was cut in half, and a profile line scan (0.5 μm) was performed along the wall. In this region, the maximum peak-to-valley height (Rz) was found to be <0.2 nm indicating a relatively smooth wall.

### Conductance Experiment

This experiment aims to explore the transport properties of condensed water within hydrophilic nanopores using the electrical conductivity as a key parameter. It was carried out under varying humidity levels RH = [60%–100%] and contact angle values Θ = [5°–60°], both of which are crucial for understanding water adsorption mechanisms.^58–62^

The experimental setup described elsewhere^34^ involves a chamber (TS102 V–F2, Instec) connected to a vapor source containing distilled water (18.2 MΩ·cm, PURELAB flex, ELGA) and a vacuum pump (XDS10, Edwards). The vacuum condition is crucial for preserving the physiochemical properties of the membrane–water interface and preventing airborne contamination. In the chamber, the membrane is positioned between an electrode and a counter electrode, as depicted in Figure 1a.

The system initially undergoes a vacuum process for the purpose of self-cleaning and is then connected to the water vapor source, which is maintained at a constant temperature of 288.15 K using a precision thermal bath (200F, Julabo) and consequently sets the actual vapor pressure inside the chamber. The vacuum pump remains connected during the whole measurement, controlling suction flow with a metering valve (NY-2M-K6, Swagelok) with a small opening of 5.5 μm to maintain low-pressure conditions and temperature stability. The temperature of the membrane is precisely controlled by a high-precision thermal stage (Mk2000, Instec) that is in direct contact with the membrane to ensure high heat conductivity.

Concerning the electrical components, the side of the membrane without Pt deposition is in contact with the bottom electrode, a thin glass plate covered with 20 nm of Pt deposited with an electron gun evaporator. The electrical connection to the Pt-covered glass is established using silver conductive paint (RS 186-3600, RS PRO) and epoxy adhesive (EA 9492, Henkel). The counter-electrode, on the membrane covered with Pt, takes the form of a metal piece with the shape of a donut, with its central portion left empty to allow water condensation on the porous membrane (see Figure 1a).

The donut has a total diameter matching that of the membrane (1.9 cm), with its inside diameter corresponding to the size of the metallic deposition (1.5 cm). It has a thickness of 2 mm and includes an additional ledge with a diameter and thickness of 0.5 mm, and this ledge is located at the internal radius of the donut and is designed to optimize pressure along the border of the metallic deposition, enhancing contact.

The regulation of RH is achieved by tuning the vapor pressure, with temperature acting as an intensive variable following the Clausius–Clapeyron relation:^63^ ln­(*P*/*P*_sat_) = −Δ*H*/(*RT*). Here, *P*, Δ*H*, *R*, and *T* are the vapor pressure, the heat of vaporization, the gas constant, and the absolute temperature. By manipulating the vapor pressure through temperature, we can precisely adjust RH, given that it is expressed as *P*/*P*_sat_ × 100. In practice, the vapor pressure inside the chamber was simultaneously monitored using a capacitance gauge (CMR362, Pfeiffer Vacuum), and saturated vapor pressure is calculated using the IAPWS formulation from 1995.^64^

### Wettability Control

Wettability properties were determined using the standard sessile drop method, which involves placing a small droplet on a substrate and analyzing the droplet’s shape to extract geometrical parameters of the liquid/solid interface, such as the contact angle Θ. For such experiments, the samples were placed within a temperature-controlled chamber, and ultrapure water droplets of approximately 2 μL in volume were deposited on the membrane’s surface. The side of the membrane set to measure Θ was the one with a 200 nm pore diameter. Side-view images of the drops were recorded with a CCD camera operating at a scan rate of 15 frames per second (fps) for subsequent contact angle measurements. The values of Θ were measured in two configurations: a membrane with Pt deposition and a membrane without Pt deposition. For both cases, the experiment was repeated at least three times with three different membranes. The values obtained were 93° ± 8° and 56° ± 4°, respectively.

Our focus of our work is on water flow within nanopores in direct contact with Al_2_O_3_. Inside the pores, the contact angle is smaller than on the Pt surface, and water will preferentially condense on the nanopore walls. From now on, when discussing wettability, we refer specifically to the contact angle with Al_2_O_3_ membrane; thus, contact angles will be measured on samples without metal deposition.

To change the wettability inside the pore, the substrates have encompassed a plasma treatment (Harrick Plasma PDC-32G at high power, 18 W applied to the RF coil with compressed air as gas). Indeed, the removal of surface impurities through ionized gas reduces the contact angle.^65,66^ Figure 2a illustrates the enhancement of hydrophilicity by displaying droplet images on the Al_2_O_3_ membrane before and after 1 min of plasma treatment.

**Figure 2:**
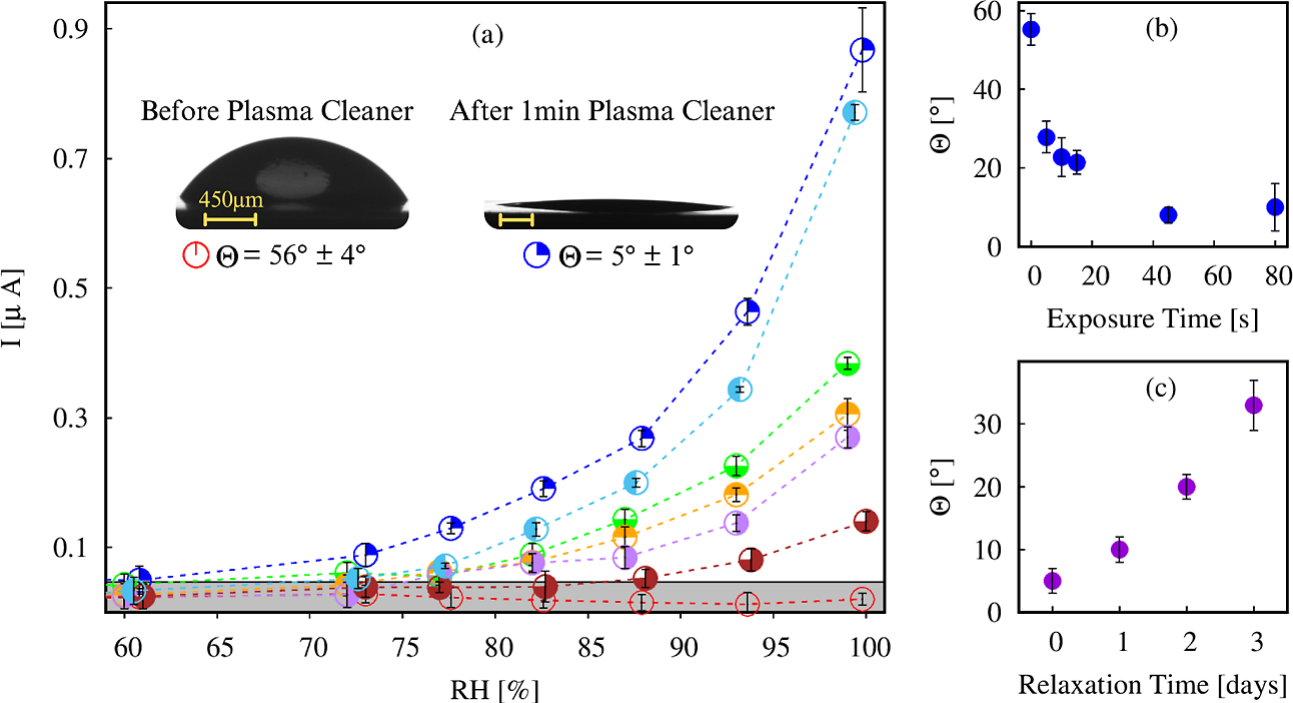
(a) Measured current (*I* [μA]) as a function of RH for various conditions. The gray band at the bottom of the graph outlines the noise region. Measured contact angles of water on the alumina membrane for each wettability were ◯: Θ = 56° ± 4°, 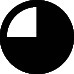: white: Θ = 28° ± 3°, ◑ ◯: Θ = 19° ± 4°, ◓: Θ = 13° ± 2°, ◒: Θ = 11° ± 1°, ◐ ◯: Θ = 9° ± 1°, and 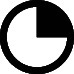: Θ = 5° ± 1°. Inset: instantaneous image (side-view) of a typical drop before and after exposure to plasma cleaner. (b) Measured contact angles (Θ degrees) of a water droplet on the alumina substrate as a function of the exposure time under plasma. (c) Measured contact angles (Θ) of a water droplet deposited on the alumina substrate as a function of the relaxation time after exposure to 1 min of plasma.

To modify the wettability of the substrate, both the plasma exposure time and the waiting duration between the treatment and sample use (relaxation time) can be varied. Studies on the temporal evolution of Θ for water on Al_2_O_3_ after plasma treatment were carried out to adjust this parameter according to our needs. Figure 2b shows a rapid decrease in Θ from 58° to 20° within 10 s of plasma treatment. However, this hydrophilic transformation is not permanent, as shown, for example, for PDMS samples, which regain their original wettability within 30 min when exposed to air.^67^

Our samples exhibit a more extended recovery period; Figure 2c illustrates the evolution of the contact angle, Θ, after a 1 min plasma treatment. It indicates that after 1 day, the sample recovers around 10% of its initial Θ value. Complete angle recovery occurs approximately 10 days after plasma exposure. To achieve different contact angles, we then utilized the two mentioned parameters: the duration of exposure to plasma (Figure 2b) and the relaxation time after a 1 min plasma cleaner treatment (Figure 2c).

Experimentally, two membranes underwent the same plasma cleaner treatment: one with Pt deposition and one without. The membrane with metallic deposition was used for conductivity measurements, and the other served as a reference for determining the contact angle (sessile drop method) on the water–Al_2_O_3_ interface. Despite the slow recovery time, to ensure that the angle measured by the reference membrane mirrors the wettability within the pores, the membrane used in the experiment was promptly introduced into the vacuum chamber after the Θ measurements.

### Conductance Measurements

A sinusoidal biased voltage with a peak-to-peak amplitude of 0.8 V at 10 Hz was applied across the electrodes, and such a selection of the voltage amplitude is to avoid electrochemical reactions. The data are sampled at a rate of 2000 Hz. The sinusoidal voltage, which avoids the accumulation of ions, will induce a current circulation along the condensed film. This current can be a consequence of the formation of hydroxyl groups at the water–Al_2_O_3_ interface facilitated by the activation of sites due to plasma cleaner treatment,^42,44,49,50,56^ among others.

The induced current was measured using an amplifier and detected on the computer with a homemade *I*/*V* converter. Both excitation and consequential signals were remotely controlled by a Python script that operated on a Digital-Analog card (NI USB-6216, National Instruments). The measured current signal is a standard sinusoid wave that shows a phase shift to the excitation stimulation, and it was further fitted by a sinusoidal function. The amplitude obtained from the fit provides us with the current response value from the water film condensed on cone walls. An example of the signals fitted is shown in Figure 1c.

To ensure the reproducibility of the detected signal, the sinusoidal voltage was cycled on and off eight times along 25 s (represented by different colors in Figure 1c), and the amplitude of the induced current was calculated as the mean of these responses. The dispersion of the values in reference of the mean is calculated by standard deviation, using the pandas library in Python. At fixed wettability, the conductance of the adsorbate liquid was systematically detected by continuously varying RH from 60% to 100%. The recovery time between each measurement was at least 2 min.

Despite a strict control on the vacuum condition, the system leakage was shown to be unavoidable.^34^ To quantify this contribution, we performed the experiment under very low humidity conditions (RH = 15%), where the observed current response is taken to be leakage conductance since the possible ions present in the system are immobile under this humidity.^34,68^ The gray band in Figure 2a delineates the region corresponding to the system’s leakage response (the noise), where the measurements are not significant.

### Water Adsorption Experiment

To track the effect of confinement on water transport, one needs to estimate the amount of water condensed on the pore walls inside the membrane. We conducted an experiment aimed at investigating the quantity of water adsorbed by the membrane in a humidity-controlled environment. To do that, we monitored the weight of the membrane thanks to a high-resolution balance (MSE225P-Sartorius) with a precision of 0.01 mg placed at various RH levels.^69^ The device of the balance includes a cubic glass chamber designed to ensure high precision of the measurements. To adjust the RH, we placed vessels with water inside the chamber. Once the chamber is sealed, water evaporates, thereby continuously increasing the RH. Quantities including RH, temperature, and weight were monitored and controlled using a combined hygrometer/thermometer (VOLTCRAFT HT-200) with a precision of 0.1% and 1°. Initially, RH was at ambient value (RH—50%), and the initial readings were disregarded to allow the system to stabilize. The actual measurements started when RH reached 60%, serving as the reference humidity value and zero point of weight measurements.

The procedure unfolded as follows: initially, a plastic surface was used as a tare, and we recorded its weight (*T*_RH_) for RH continuously increasing from 60% to 80%. Then, the surface was dried by using compressed air. A second experiment was conducted later, this time with five membranes deposed on the plastic surface (*W*_RH_). Before starting to record *W*_RH_, we weighed the plastic surface without the membranes. We attached the membranes only if the plastic surface weight matched with the first value measured in the previous experiment. The mass of water adsorbed at RH by one membrane, Δ*W*_RH_, is calculated as the difference in weight between the two scenarios divided by 5, minus the mass of water adsorbed at RH = 60%, which serves as the initial measurement point.

1 $$\Delta W_{RH}=\frac{W_{RH}-T_{RH}}{5}-\Delta W_{60\%},,RH\in \left[60\%,80\%\right]$$

This procedure was repeated at least four times for two extreme cases of wettability: when the membranes experienced (Θ = 8° ± 3°) or not (Θ = 58° ± 5°) 1 min of plasma exposure. All experiments were conducted at a temperature *T* ≈ 300 K, resulting in Δ*W*_60%_ of 0.28 ± 0.02 g for the contact angle Θ = 8° ± 3° and 0.26 ± 0.02 g for Θ = 58° ± 5°. The maximum RH we could achieve, RH = 80%, results from the limited size of the balance chamber, which can only hold a limited number of water vessels.

## Results and Discussion

Electrokinetic measurements were performed for seven different wettabilities with RH values ranging from 50% to 100%, and they are summarized in Figure 2a. Figure 2b,c depicts the contact angles obtained after different periods under plasma treatment and by relaxation time after 1 min of plasma exposure (check the details in the subsection wettability control). Analyzing the curves on Figure 2a, we observe a noisy background from the membrane before plasma cleaning, suggesting that there is no dissociative adsorption. Subsequently, after plasma cleaning, as the hydrophilicity increases, so does the current response. In addition, the current also increases when the RH rises.

The above findings can be intuitively understood based on previous reports. Increasing wettability increases the thickness of the adsorbed water layer, as indicated in the research of Guo et al. for metallic materials.^70^ For water adsorption and transport on flat Al_2_O_3_ surfaces, it has been established that the positive correlation between the electrokinetic current and the humidity is associated with the increasing thickness of adsorbed water layers.^71^ Regarding our results with porous Al_2_O_3_ membranes, the higher induced current with increasing wettability and humidity may also be related to adsorption properties, such as the thickness of the water film adsorbed inside the pores. Our objective of this study is then to determine the thickness of the adsorbed layer as a function of RH.

### Water Adsorption

In hydrophilic nanopores with diameters over 8 nm, sorption happens from the walls toward the center (radial-pore-filling mode).^72,73^ The critical humidity for the transition from multilayer adsorption to capillary condensation (supersaturation) varies with the pore diameter. For pores larger than 100 nm, the effect of confinement is negligible, and this transition starts from the saturated point RH = 100%.^74^ This is indeed the case for our nanoporous membrane, and then, the pore-filling process follows a multilayer scenario, where the thickness of the water film increases with humidity.^75^

There are several models to study the adsorption of gases by surfaces. The Brunauer–Emmett–Teller (BET) model^76^ is often used in the case of porous surfaces to describe multilayer adsorption, but it only works well in the range of relatively low partial pressures (*P*/*P*_0_) typically between 0.05 and 0.35,^77^ which is out of our experimental range. In our experimental range, the Polanyi theory^57^ appears more relevant, which considers the interactions between the gas molecules and the surface of the adsorbent materials via the van der Waals attraction. Even though the van der Waals interaction theory does not account precisely for the hydrophilic interactions,^56^ it is able to provide a reasonably effective water–surface interaction. The application of this approach for water and porous Al_2_O_3_ interactions has already been reported.^78^

The Polanyi equation, which then directly links the water adsorbed density with the RH, is expressed as^79^

2 $$\Gamma =\frac{1}{V_{m}^{L}}{\left(\frac{C}{RT\;\log\left(1/RH\right)}\right)}^{1/3}-\frac{D_{0}}{V_{m}^{L}}$$

where Γ is the number of moles of water adsorbed per unit of surface, which can be expressed as the ratio between the thickness of the adsorbed layer and the molar volume of the liquid, Γ = *h*_RH_/*V*_m_^L^, *D*_0_ is the molecular radius of the adsorbate (gas), *V*_m_^L^ is the molar volume of the liquid, and *C* = πρ_A_ *C*_AB_/3 is a constant derived from Gibbs free energy for the van der Waals interaction (A is the adsorbate and B is the adsorbent). *R* is the constant of perfect gases and *T* = 297 K the temperature. For water, *V*_m_^L^ = 18 × 10^–6^ m^3^/mol and *D*_0_ = 0.135 nm.

We will use the isotherm eq 2 to determine the amount of adsorbed water as a function of humidity in our experiments (see Experimental Section for details). We have to stress here that our measurements of water adsorption as a function of humidity are not absolute but relative to the value at RH = 60%, and we then need a model to get the absolute value of adsorbed water.

In detail, after measuring the mass adsorbed by the membrane Δ*W*_RH_, we can deduce the water layer thickness *h*_RH_ formed inside the pores, if we assume it is homogeneously distributed. The RH was varied between 60% and 80%, ensuring a multilayer adsorption regime. We remind that the experiment RH = 60% is taken as a reference point, or point zero of the measures. Thus, the water layer thickness *h*_RH_ is the sum of the thickness at this reference point, RH = 60%, plus the thickness deduced from the balance measurements *h*′ = Δ*W*_RH_/2π*r*_np_ *LN*_Ap_ρ_w_. This reads:

3 $$h_{RH}=h_{60\%}+h^{\prime },RH=\left[60\%\text{−}80\%\right]$$

with the water density ρ_w_ = 0.997 × 10^–21^ g/nm^3^, *r*_np_ = 75 nm the nanopore average radius, *L* = 55 μm the membrane thickness, and *N*_Ap_ ≈ 2.7 × 10^9^ the number of the pores in the entire membrane (including the section without Pt deposition).

Along with *h*_RH_, the adsorption density Γ has two components: Γ_60%_ at RH = 60%, which can be derived using eq 2, which sets the background, and the additional adsorption when RH > 60%, Γ^′^ = *h* ^′^/*V*_m_^L^. Figure 3a shows the number of molecules per area, Γ′, as a function of RH for two cases with and without 1 min’s plasma treatment; the points are deduced from experimental data, and the line represents the fit described by Γ′ = Γ – Γ_60%_ (eq 4), with *C* as the fitting parameter:

4 $$\Gamma^{\prime }=\frac{1}{V_{m}^{L}}{\left(\frac{C}{RT\;\log\left(1/RH\right)}\right)}^{1/3}-\frac{1}{V_{m}^{L}}{\left(\frac{C}{RT\;\log\left(1/0.6\right)}\right)}^{1/3}$$

**Figure 3:**
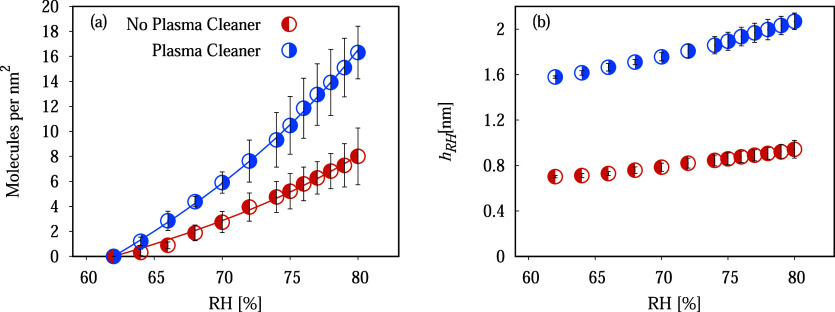
(a) The number of molecules adsorbed per unit of surface (Γ′) taking RH = 60% as reference point for RH as a function of humidity in the range of [60%–80%]. The data is an average of five experiments, and error bars illustrate the resulting dispersion. The line is a fit using eq 4. (b) Water layer thickness (*h*_RH_) condensed inside the nanopores using expression 3 as a function of humidity in the range RH = [60%–80%].

The values of *C* obtained from the fit are *C*_n_ = 690 J nm^3^/mol and *C*_pc_ = 5930 J nm^3^/mol for the cases without and with plasma activation, respectively. This parameter is related to the strength of van der Waals forces between the adsorbent and adsorbate and is proportional to the Hamaker constant *A* = 3π*C*ρ_A_, which quantifies the strength forces between particles or surfaces. By evaluating this Hamaker constant from *C*_n_ and *C*_pc_, we obtain *A*_n_ = 3.6 × 10^–19^ J and *A*_pc_ = 3.1 × 10^–18^ J, in fair agreement with values reported from the literature.^80^ This indicates that the Polanyi theory of adsorption, even though using van der Waals interactions, which are not accurate for giving the hydrophilic interaction with water, leads to the correct water–surface interaction according to our macroscopic data from the water adsorption experiment.

Moreover, the Hamaker constant for the plasma-activated case is larger than the nonactivated case, (*A*_pc_ > *A*_n_), indicating that the adsorption force between gas molecules and the surface has increased after the plasma treatment. As previously mentioned, the plasma cleaning process uses ionized gas to treat the surface with plasma ions. After this process, the creation of active sites enhances surface reactivity^81^ and adsorption forces, potentially increasing the Hamaker constant. This also promotes dissociative adsorption at the Al_2_O_3_/water interface. As observed earlier, there is no measured ionic current when the membrane has not been treated with a plasma cleaner (Figure 2a). Using the value found of the constant C, we can then estimate the absorbed thickness *h*_60%_ at RH = 60%. For the case without the plasma procedure, the thickness obtained is *h*_60%_ ≈ 0.7 nm, and for the case with the lowest contact angle, *h*_60%_ ≈ 1.6 nm. In Figure 3b, we show the absolute thickness *h*_RH_, using the values found for *h*_60%_ and *h*′.

### Ionic Transport

Moving forward, we estimate the conductivity and conductance in the adsorbed film to investigate transport properties in our membrane. Therefore, our focus will be on the case treated with the longest plasma (Θ = 5° ± 1°, Figure 2a), as it shows a higher current response and reactivity. We inspect the data on the current *I* for this wettability (Figure 4a), and we analyze the conductance trend (*G*) using the following equation:

5 $$G=\frac{I}{V}$$

where *V* is fixed at 0.8 V. Since we have the values of *G* and *h*_RH_ for different RHs, we can directly correlate them as shown in Figure 4c. The data point corresponds to experimental data using this procedure, which is obtained from the thickness and current values measured for precisely the same RH. Solid lines correspond to values obtained from extrapolations made within the RH range of 70% to 85%, the range in which experimental measurements for both *I* and *h*_RH_ coexist. It consists of a quadratic fit for *I*(RH) measurements and a linear fit for *h*_RH_(RH) data, as reported in Figure 4a,b.

**Figure 4:**
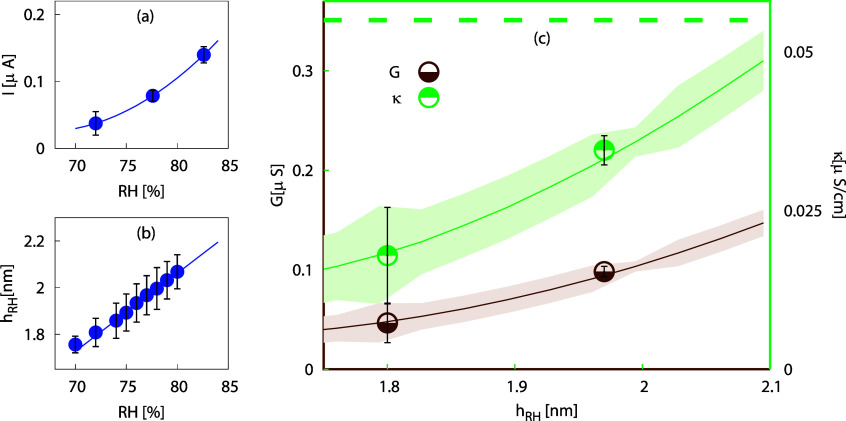
(a) Current measured for Θ = 5° ± 1° within the RH range of [70%–85%], corrected by subtracting the noise value (0.051 μA). The line represents an extrapolation using the quadratic equation *f*(*x*) = *ax* ^2^ + *bx* + *c*, where *a* = 4.6 × 10^–4^ μI per %^2^, *b* = −6 × 10^–2^ μI per %, and *c* = 2 μI. (b) Water layer thickness within the same RH range. The line is a extrapolation using a linear function *f*(*x*) = *ax* + *b*, where *a* = 3 × 10^–2^ nm per % and *b* = −0.6 nm. (c) Left *y*-axis, brown: estimated conductance as a function of the number of monolayers for the case Θ ≈ 5°. The line was obtained using eq 5, by extrapolating current *I* (a) and thickness *h*_RH_ (b). Data points were obtained from the same equation directly correlating the *I* (a) and *h*_RH_ (b) data points under the same humidity. Right *y*-axis, green: estimated conductivity as a function of the number of monolayers also for Θ ≈ 5°. The line was obtained by the same extrapolation (a,b), but using eq 6 for *L* = 55 μm, *S* = 2π*r*_np_ × *h*_RH_, *r*_np_ = 75 nm, and *N*_p_ = 1.7 × 10^9^. The points were obtained using the data points from (a,b) at the same RH, using eq 6. The dashed line illustrates the conductivity value of the bulk distilled water κ_w_ = 5.5 μS/m. The shaded areas behind the lines indicate extrapolation errors, which include errors from existing data points and an average error estimated using the mean of all measured errors, applied to the extrapolated points.

As the thickness increases in Figure 4c (left axis), the conductance also increases. This correlation may be a consequence of the fact that in the initial layers, the interaction forces between the liquid and the wall are very strong. As the liquid fraction expands, these forces decrease with the distance from the surface, thus facilitating conductance.

To better understand this result, we further study the trend of conductivity κ by extrapolating *I* and *h*_RH_ as we did for *G*, using the relation

6 $$\kappa =G\times \frac{L}{SN_{p}}$$

where the cross-sectional area of the conducting water layer is given by *S* = 2π*r*_np_ × *h*_RH_, and *L* is the distance between the electrodes (length of pores/membrane thickness). In Figure 4c (right axis), the resulting conductivity trend is presented, along with data points, and the dashed line represents the conductivity of bulk water, which is κ_b_ = 5.5 μS/m. Note that we neglected here so-called surface conductivity,^12^ due to the low reactivity of Al_2_O_3_ with water at these pH.^82^ Moreover, this hypothesis is justified by the fact we observed a conductivity in the water film even lower than the water bulk conductivity, showing then a negligible contribution of added contributions such as the surface one.

The trend line indicates that conductivity increases with humidity but remains lower than that of bulk water for the RH values studied here. This result is remarkable, as the conductivity is expected to be inversely proportional to *h*_RH_. One first can note that the conductivity is, by definition, in ionic solution linked to the ion mobility noted μ. Thanks to the Einstein relation, the ion mobility and the diffusion coefficient are linked by

7 $$D=\mu k_{B}T=k_{B}T/\left(6\pi \eta R_{ion}\right)$$

As the ionic current is reduced when the water thickness is small, so is the ionic mobility. The simplest continuous model to account for this effect is to divide the water into two layers, one with zero mobility, the so-called “stagnant” layer, and one above, with the bulk mobility. This approach has been proposed recently for water in the vicinity of SiO_2_ (see ref 34).

Based on our results showing lower conductivity compared to the bulk value, we propose an analogous interpretation, pointing out that a fraction of the film condensed on nanopore walls may consist of a stagnant layer with a zero mobility. We can estimate the thickness of the stagnant layer, *h*_s_, by calculating the difference between the total thickness, *h*_RH_, and the mobile portion, using the conductance values from the points (Figure 4). The mobile portion is assumed to have the same conductivity as bulk water, κ_b_, and the experimentally obtained conductance, *G*, is attributed to this portion. By applying these values to eq 6, we determine *h*_s_ by subtracting the mobile part from the total film

8 $$h_{s}=h_{RH}-\frac{GL}{\kappa_{b}2\pi r_{np}N_{p}}$$

By averaging the stagnant thickness based on measurements shown in Figure 4c, we determine a stagnant thickness of *h*_s_ = 0.97 ± 0.34 nm. Since a water monolayer is typically about 0.3 nm thick, it suggests approximately three stagnant monolayers at the interface. This value is in good agreement with literature data, suggesting that the range of influence of the Al_2_O_3_ surface inducing an ordered molecular arrangement was observed to extend about 1 nm from the surface.^83^

Indeed, Wang et al.^56^ suggest that strong hydrogen bonds at the water/α-Al_2_O_3_ interface lead to ordered water structures, slowing down the translational and rotational dynamics of interfacial water compared to bulk water. As the thickness increases, the behavior and conductivity approach those of bulk water. A similar phenomenon was reported previously.^71^ Other works suggest that this stagnant layer is characterized by subdiffusive motion^84^ due to the well-structured organization of water molecules near the surface, indicating an “ice-like” configuration.^85,86^ In films thicker than four to five monolayers, the water may recover its bulk properties.^87^

One can discuss the link between water structuring and the reduced conductivity. Indeed, two possible mechanisms can account for the reduced mobility (eq 7): (i) the viscosity of water in this zone is diverging due to this ordering (the water behaves as a solid); (ii) the ion motion is inhibited due to the specific water structure, even if the water viscosity remains the bulk one. We cannot decipher between the two proposed mechanisms, which would require some mass transport measurements at the nanoscale. However, our findings show for the first time some consequences for macroscopic ionic transport of water ordering near Al_2_O_3_.

Similarly, for SiO_2_ interfaces, three layers with an “ice-like” structure have been observed,^85^ although a single layer of stagnant water was reported.^34^ These differences between Al_2_O_3_ and SiO_2_ have been documented, the Al_2_O_3_ surface interacting more with polar water molecules and then influencing ordering at a larger scale in the liquid water.^82^

Thus, the thickness of the stagnant layer *h*_s_ measured in our study aligns with previously observed values at the water/solid interfaces. Despite the increased adsorption force due to post-plasma treatment, which, as previously calculated, increases approximately 10-fold according to the Hamaker constant, we do not observe a thicker layer *h*_s_. Therefore, despite the stronger binding energy, it appears that the range of “ice-like” structure at the solid/liquid interface does not change significantly.

## Conclusions

In this study, we probed how the current response of water films inside nanopores changes with variations in two factors related to water adsorption: the material’s wettability and the RH. One parameter, wettability, pertains to the material, while the other, RH, pertains to the environmental conditions. A nanoporous membrane made of oxidized aluminum, Al_2_O_3_, and naturally hydrophilic was used in the research, and its wettability was tuned by plasma treatment. We noted an increase corresponding to higher RH and wettability, implying a correlation with the amount of adsorbed water or the pore filling thickness.

To quantify the amount of water adsorbed at different wettability levels, we used a balance to measure the quantity of water adsorbed by the membrane under varying RH conditions. Applying Polanyi theory to describe the physics of this isotherm, we found that the adsorption energy, measured by the Hamaker constant, increased approximately 10-fold after 1 min of plasma cleaning compared to untreated cases. Regarding conductivity for the most hydrophilic case, which exhibited a stronger current response, we found that the values were lower than that of bulk distilled water (κ_b_), approaching it as RH increases. This behavior is attributed to interactions with pore walls, which induce ordered stagnant layers near the interface. Our findings suggest the presence of approximately three stagnant monolayers. This value is in accordance with previous studies, indicating that the increase in the adsorption energy does not lead to thicker immobile layers. Consequently, we can see that higher hydrophilicity enhances transport properties due to the increase in mobile layers. Our findings are important for understanding the properties of water films adsorbed by nanopores under different wettability conditions and environmental humidities and contribute to the development of future nanofluidic devices at the scale of a few nanometers.
